# The interactions of physical activity, exercise and genetics and their associations with bone mineral density: implications for injury risk in elite athletes

**DOI:** 10.1007/s00421-018-4007-8

**Published:** 2018-10-30

**Authors:** Adam J. Herbert, Alun G. Williams, Philip J. Hennis, Robert M. Erskine, Craig Sale, Stephen H. Day, Georgina K. Stebbings

**Affiliations:** 1 Department of Sport and Exercise, School of Health Sciences, Faculty of Health, Education and Life Sciences, Birmingham City University, Birmingham, UK; 2 Sports Genomics Laboratory, Manchester Metropolitan University, Cheshire Campus, Crewe Green Road, Crewe, CW1 5DU UK; 3 Musculoskeletal Physiology Research Group, Sport, Health and Performance Enhancement Research Centre, Nottingham Trent University, Clifton Lane, Clifton, Nottingham, NG11 8NS UK; 4 Research Institute for Sport and Exercise Sciences, Liverpool John Moores University, Byrom Street, Liverpool, L3 3AF UK; 5 Institute of Sport, Exercise and Health, University College London, Tottenham Court Road, London, W17 7HA UK; 6 Department of Biomedical Science & Physiology, School of Sciences, Faculty of Science and Engineering, University of Wolverhampton, Wolverhampton, UK

**Keywords:** Genetics, Bone, Exercise, Polymorphism, Running, Fracture

## Abstract

Low bone mineral density (BMD) is established as a primary predictor of osteoporotic risk and can also have substantial implications for athlete health and injury risk in the elite sporting environment. BMD is a highly multi-factorial phenotype influenced by diet, hormonal characteristics and physical activity. The interrelationships between such factors, and a strong genetic component, suggested to be around 50–85% at various anatomical sites, determine skeletal health throughout life. Genome-wide association studies and case–control designs have revealed many loci associated with variation in BMD. However, a number of the candidate genes identified at these loci have no known associated biological function or have yet to be replicated in subsequent investigations. Furthermore, few investigations have considered gene–environment interactions—in particular, whether specific genes may be sensitive to mechanical loading from physical activity and the outcome of such an interaction for BMD and potential injury risk. Therefore, this review considers the importance of physical activity on BMD, genetic associations with BMD and how subsequent investigation requires consideration of the interaction between these determinants. Future research using well-defined independent cohorts such as elite athletes, who experience much greater mechanical stress than most, to study such phenotypes, can provide a greater understanding of these factors as well as the biological underpinnings of such a physiologically “extreme” population. Subsequently, modification of training, exercise or rehabilitation programmes based on genetic characteristics could have substantial implications in both the sporting and public health domains once the fundamental research has been conducted successfully.

## Introduction

Low bone mineral density (BMD) is established as a primary predictor of osteoporotic risk and can have substantial implications for athlete health and injury risk in the elite sporting environment. BMD is a highly multi-factorial phenotype influenced by diet, hormonal characteristics as well as physical activity (Darling et al. 2009; Pluijm et al. 2001). Physical activity/exercise reportedly accounts for up to 30% of the variability in BMD (Valdimarsson et al. 1999) although the exact contribution of physical activity to BMD remains unclear and requires further exploration across various population groups. Following physical activity, osteocytes detect shape and volume changes to increase or decrease the liberation of specific bone mediators, which consequently influences bone formation and resorption (Nakashima et al. 2011). Consequently, athletic populations tend to possess higher BMD than non-athlete counterparts. Training and competition in weight-bearing sports that comprise high strain rates and peak-force loading characteristics on bone result in enhanced total or site-specific BMD as shown across a number of sports (Torstveit and Sundgot-Borgen 2005). This principle, however, can be more complex in sports that are associated with low body mass or reduced energy availability, such as endurance running, where low BMD and stress fractures can be observed (Pollock et al. 2010; Loucks 2007). Additionally, the volume of physical activity completed in childhood and the age at which an athlete may have started their sport may also have implications for BMD across the lifespan. Generally, childhood and the pre-pubertal years are considered a key period for bone accretion (Weaver et al. 2016). A large volume of research into the effect of physical activity on BMD and/or osteoporosis has been completed although limited investigations exist regarding certain athletic populations such as endurance runners. Moreover, many studies have used questionnaires to assess physical activity level, which can lack accuracy or reliability (Prince et al. 2008) and thus, the exact contribution of physical activity remains unclear and requires further exploration across population groups.

A large genetic component to BMD also exists, with heritability of BMD suggested to be 50–85% depending upon anatomical location (Ralston and Uitterlinden 2010). Knowing the genetic variants associated with BMD could have substantial implications for future research, as well as application and rehabilitation management in both the public health domain and elite sporting environment. For example, accuracy of fracture risk classification was improved by 7–10% in osteopenic patients by adding an early genetic risk score (Lee et al. 2014) whilst modifying training programmes based on genetic characteristics reduced injury rates in endurance athletes (Goodlin et al. 2015). Practical application using genetics, however, is currently very restricted due to limited evidence on proposed candidate genes associated with BMD. Over 66 genetic loci have been associated with dual-energy X-ray absorptiometry (DXA)-derived BMD or fracture via genome-wide association studies (GWAS) thus far (Hsu and Kiel 2012; Estrada et al. 2012) and this number will continue to increase. Additionally, many of the previously discovered candidate genes have had little or no replication through further study, which means only a very small number can be confidently suggested to have an association with BMD (Hsu and Kiel 2012). The biological function or involvement with bone metabolism of 30 of these has also yet to be elucidated and only seven of the 66 have been associated in candidate gene studies previously or positively replicated afterwards (Hsu and Kiel 2012) although some have received no further study as of yet. Studies so far have only elucidated a fraction of BMD variance and thus, some of the unexplained heritability is likely due to a number of factors such as gene–environment interactions (Ackert-Bicknell and Karasik 2013). This could apply most strongly to certain populations such as athletes, due to the substantial influence of physical activity on BMD and a likely gene–mechanical loading interaction.

The genetic influence on BMD and the relationship with physical activity has not been explored extensively. In vitro studies have shown substantial alteration in gene expression following mechanical loading (Mantila Roosa et al. 2011), whilst a small number of candidate genes have reported physical activity interactions in children (Mitchell et al. 2016). A small number of investigations have also been completed in athletic populations across a number of different bone phenotypes. For example, higher total BMD in weight-bearing athletes than controls was observed in the FF (7.7%) and Ff (6.9%) but not ff (1.8%) genotypes of the vitamin D (1,25-dihydroxyvitamin D_3_) receptor (*VDR*) FokI rs2228570 polymorphism, whilst lower total BMD was only observed in the FF (− 4.5%) genotype when comparing swimmers with a control group (Nakamura et al. 2002b). Additionally, variants in the purinergic receptor P2X7 (*P2RX7*), human TNF receptor superfamily member 11a (*TNFRSF11A*) and sclerostin (*SOST*) genes have been associated with stress fracture in elite athletes (Varley et al. 2015, 2016, 2017). Substantial further study is needed on candidate genes associated with BMD and other phenotypes such as stress fracture, as well as greater exploration of genes that may interact with physical activity and the implications this would have for BMD and wider application in public health and elite sport.

Therefore, the aims of this narrative review are to (1) provide a critical review of the current literature on the influence of physical activity on BMD, particularly in athletic populations such as endurance runners; (2) provide an overview of genetic associations with BMD and highlight studies that have assessed this association in athletic populations; and (3) explore gene–BMD–physical activity interactions and identify future applications these might have in both the public health domain and elite sporting environment.

## Bone mineral density (BMD)

Peak bone mass is a function of bone size and volumetric BMD (Leonard and Bachrach 2012) and thus, is the amount of bony tissue present following skeletal maturation, which can have a substantial influence on osteoporotic risk in later life (Bonjour et al. 1994). BMD is defined as the ratio of mass to the area or volume of bone, which is known as areal (g/cm^2^) or volumetric (g/cm^3^) BMD, depending upon the measurement methodology used (Ott et al. 1997). BMD is considered the primary predictor of osteoporotic fracture, although it is important to note other factors when assessing clinical risk (Cranney et al. 2007). BMD accounts for 60–65% of the variance in bone strength so other factors such as bone geometry, collagen properties as well as trabecular and cortical microarchitecture are also important determinants of bone strength (Schoenau et al. 2002; Fonseca et al. 2014; Cheung et al. 2016).

**Fig. 1:**
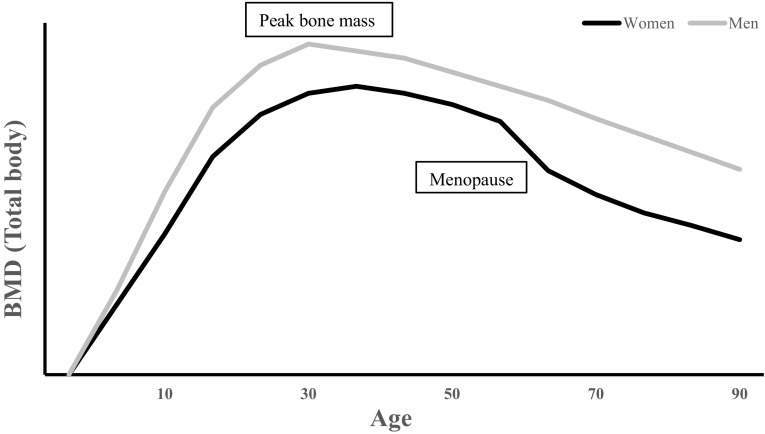
Schematic representation of typical age- and sex-related loss of BMD in men and women

Bone mass is regulated by the activity of osteocytes in response to a number of stimuli, such as disuse, matrix damage or hormone deficiency (Atkins and Findlay 2012) and the actions of osteoblasts and osteoclasts, which are important for bone formation and resorption. Disproportionate activity rates of these bone cells, for instance, greater net osteoclastic than osteoblastic activity, can cause bone loss, as observed in ageing (Martin and Sims 2005). Approximately 85–95% of peak bone mass is attained around late adolescence (Henry et al. 2004; Walsh et al. 2009). After peak bone mass is reached, BMD loss occurs as we age (Fig. 1) and the rate of loss plays an important role in bone health and the development of related conditions, such as osteoporosis (Hernandez et al. 2003). BMD deterioration varies between individuals as well as anatomical sites, with yearly rates of decline after the age of 25 years at the distal radius, distal tibia and lumbar spine reportedly 0.40%, 0.24% and 1.61%, in women and 0.38%, 0.40% and 0.84%, in men. Additionally, men and women experience 42% and 37% of trabecular bone loss as well as 15% and 6% of cortical bone loss before the age of 50 years (Riggs et al. 2008). Similar to the ability to enhance peak BMD with lifestyle choices, it is possible to slow the inevitable decline in BMD with ageing using preventative measures via lifestyle modification. Some of these factors include not smoking (Law and Hackshaw 1997), maintaining a healthy dietary intake (Darling et al. 2009) and relatively high physical activity level (Pluijm et al. 2001; Krall and Dawson-Hughes 1993).

### BMD and physical activity

Quantifying the relative contributions of physical activity and other determinants to BMD remains difficult. Exercise/physical activity reportedly accounts for up to 30% of the variability in total BMD (Table 1), emphasising that the contribution of physical activity to BMD remains unclear and requires further exploration across various population groups.

**Table 1:** Contribution of physical activity to BMD

Population	BMD determinant	Variability in BMD	References
Icelandic women aged 16–20 years	Lean mass and physical exercise	30%	Valdimarsson et al. (1999)
Men and women aged 20–25 years	Sports activities	10.4%—men < 1%—women	Neville et al. (2002)
Pre-menopausal women aged 35–37 years	Member of sports club completing persistent weight-bearing activity in adulthood	5–19%	Barnekow-Bergkvist et al. (2006)
European Caucasian men aged 65–80 years	High-impact unilateral training programme on one leg (EL) in comparison with the other leg (CL)	1.6% net gain in femoral neck between EL and CL	Allison et al. (2015)
Men and women aged 20–54 years	Physical activity level	Active women and men had 2.7–4.6% and 1.9–3.0% higher BMD, respectively, than sedentary counterparts	Morseth et al. (2010)
Men aged 17–20 years	Physical activity habits	10.1%	Pettersson et al. (2010)

Initially proposed by Wolff’s law and Frost’s mechanostat theory, bone adapts or remodels in response to the forces or demands placed upon it (Frost 1990). This mechanotransduction is completed through four steps: mechanocoupling, biochemical coupling, signal transmission and effector cell response (Duncan and Turner 1995). Bone metabolism is regulated via specific pathways, such as the receptor activator of nuclear factor κB/receptor activator of nuclear factor κB ligand/osteoprotegerin (RANK/RANKL/OPG), Wnt signalling and purinergic signalling pathways, through initiation of osteoblastic or osteoclastic activity (Tyrovola and Odont 2015). Following physical activity, osteocytes detect shape and volume changes to increase or decrease the liberation of these bone mediators, which consequently influences bone formation and resorption (Nakashima et al. 2011). This notion has been observed in numerous populations including children, adults and older adults, with those who complete a large volume of physical activity/exercise possessing greater BMD, strength and muscle mass (Chilibeck et al. 1995; Slemenda et al. 1991; Beck and Snow 2003; Warburton et al. 2006). The point in time when this physical activity occurs may also influence bone development and bone mass, potentially resulting in lifetime benefits for skeletal health (Gunter et al. 2012). Generally, weight-bearing activity in childhood has been shown to increase total body BMD in adolescents and children (Weeks et al. 2008; Heidemann et al. 2013), as well as demonstrate a continued benefit into adulthood at key sites such as the femoral neck and lumbar spine (Strope et al. 2015). Tveit et al. (2013) reported that exercise-associated high BMD in 46 young male athletes (mean age = 22 years) was preserved three decades after retirement and cessation of high volumes of physical activity. Similarly, ex-professional baseball players in their ninth decade of life retained more than half of the throwing-related benefits in bone size and a third of the throwing-related benefits in bone strength observed in current professionals (Warden et al. 2014).

Some studies have suggested that activity completed in the pre-pubertal stage is the most favourable to instigate bone development due to the elevated levels of growth hormone present at this time (Bass et al. 1998). Growing bone has an enhanced capability to respond to increased mechanical loading and thus initiate greater structural adaptations to this stimulus, compared to adult bone (Bass et al. 1998). This notion of an optimal period or “window of opportunity” for exercise-induced bone development could be important in improving bone health by maximising peak bone mass attainment during this time (Bass et al. 1998) and therefore, delaying the onset of age- or menopause-related osteoporosis (Santos et al. 2017). Despite this, Behringer et al. (2014) completed a meta-analysis suggesting that weight-bearing activities in childhood and adolescence had no significant influence on BMD in adulthood. The authors based their conclusion, however, on 27 studies out of a possible 109 completed before 2012 and suggest their findings might have been skewed as a result. Therefore, the overall consensus, as outlined by the National Osteoporosis Foundation’s recent position statement, is that the best evidence suggests a positive effect of physical activity during late childhood and pre-pubertal years and this is a key period for bone accretion (Weaver et al. 2016).

The ability to complete studies that are both longitudinal and valid accounting for accurate measurement of activity (i.e. quantifying intensity in relation to the bone-loading forces experienced) is extremely problematic. Many investigations have used self-report activity questionnaires rather than more direct measurements, such as via accelerometers or pedometers (Ondrak and Morgan 2007). Self-report questionnaires rely upon recall and response bias; correlations between self-report and direct measurement of physical activity have been reported as low-to-moderate, ranging from − 0.71 to 0.96 (Prince et al. 2008). Whilst accelerometers are capable of objectively quantifying activity level, this is still an estimation limited by validity, reliability and calibration concerns (Troiano et al. 2014) as well as being unable to provide direct measurement of the stimulus applied to any particular bone or the skeleton as a whole. Furthermore, there has been much methodological variance in studies exploring this topic, such as participant characteristics and sample size, the differing methods used to measure physical activity and types of physical activity/exercise completed in the training intervention. These factors make it difficult to draw conclusions on the exact influence of physical activity on BMD and may explain the large variability in the extent of the skeletal response to loading reported in intervention studies. For reviews on this topic, see Warburton et al. (2006) and Ondrak and Morgan (2007).

Quantifying the optimum amount of physical activity for bone health is both difficult and complex when considering all of the potential confounding variables. Research has suggested that the current US Department of Health and Human Services and UK Chief Medical Office physical activity guidelines do not allow maximisation of BMD potential (Whitfield et al. 2015). Additionally, the type of physical activity may also be important for optimising BMD. Habitual levels of high, but not moderate or light, physical activity was positively related to BMD in adolescents (Deere et al. 2012) as well as in older adults (Hannam et al. 2017). However, high impacts in adolescents were classed as > 4.0*g* but only > 1.5*g* in the older adults. Thus, the impact threshold required to combat bone loss is likely to be lower in older adults but higher *g* forces may be required to stimulate acquisition during peak attainment in childhood (Tobias 2014), which adds further complexities to understanding the influence of physical activity on bone health. Therefore, due to the difficulty of quantifying physical activity and the large number of determinants of BMD, investigating the influence or association of physical activity on BMD is challenging. Using homogenous cohorts that are known to be undertaking similar amounts of physical activity, such as athletic populations, can somewhat alleviate this issue.

### BMD in athletic populations

Physical activity can be defined as any movement implemented by skeletal muscle that results in energy expenditure, whereas exercise refers to physical activity that is planned, structured and repetitive with an aim to maintain or improve a physical fitness component (Caspersen et al. 1985). Therefore, athletic populations who complete large volumes of exercise also tend to possess higher BMD and bone mass than non-athletic individuals via the loading adaptation mechanisms mentioned above (Chilibeck et al. 1995). However, the loading characteristics of different sports vary, thus the BMD of athletes partaking different sports or disciplines also varies, particularly between different anatomical sites (Mudd et al. 2007; Bennell et al. 1997). One of the earliest applied studies investigating BMD of athletes competing in different sports showed significantly higher total and site-specific BMD in volleyball players in comparison with gymnasts, swimmers and non-athletic controls, although the BMD of the gymnasts was significantly higher than the other two groups (Fehling et al. 1995). This emphasises that physical activity/exercise, which expresses higher impacts through increased strain rates and high peak-force loading characteristics, as can be expected of volleyball players, results in enhanced total or site-specific BMD as shown across of a number of sports (Table 2).

**Table 2:** BMD variation across different sports

Population	Sport	BMD variation	References
300 Norwegian female elite athletes (national level at senior or junior) 300 non-athletic controls	66 sports	3–20% higher BMD than controls. 3–22% higher BMD in high-impact sports compared to medium- or low-impact sports	Torstveit and Sundgot-Borgen (2005)
15 elite male athletes 15 non-athletic controls	Volleyball	14% and 24% higher BMD at the lumbar spine and femoral neck, respectively, in volleyball players in comparison with non-athletic controls	Calbet et al. (1999)
14 state-level female athletes 18 non-athletic controls	Netball	7.8%, 17.3% and 14% higher total body, hip and lumbar spine BMD in the netballers in comparison with the controls	Chang et al. (2013)
50 male highly trained athletes 12 non-athletic controls	12 judokas 14 karate athletes 24 water polo players	Control group total body BMD (1.27 g/cm^2^) was significantly lower than the judo (1.40 g/cm^2^) and karate (1.36 g/cm^2^) group but no different to the water polo athletes (1.31 g/cm^2^)	Andreoli et al. (2001)
59 competitive Finnish female athletes 25 physical active individuals 25 sedentary individuals	27 dancers 18 squash players 14 speed skaters	Squash players had significantly higher BMD at the lumbar spine (13%), femoral neck (16.8%), proximal tibia (12.6%) and calcaneus (18.5%) in comparison with the sedentary group. Aerobic dancers also had significantly higher BMD at the loaded sites in comparison with the sedentary group, ranging from 5.3 to 13.5%	Heinonen et al. (1995)
60 athletes 15 non-athletic controls	15 runners 15 swimmers 15 triathletes 15 cyclists	Runners had significantly higher total body, femoral neck and leg BMD than controls and swimmers as well as higher leg BMD than cyclists	Duncan et al. (2002)

In endurance runners specifically, most studies have shown a higher BMD than control populations, particularly at the primary loading sites (tibia, femoral neck, calcaneus), although this is not always the case due to other variables, such as low energy availability (Scofield and Hecht 2012). However, endurance runners tend to have lower BMD than athletes from other weight-bearing sports, such as sprinters or gymnasts, where forces applied to bone are more likely to be varied in magnitude and directions (Scofield and Hecht 2012). Master athletes over the age of 65 years who are still competing in running events have been shown to possess higher BMD than non-active counterparts (Velez et al. 2008). Furthermore, former elite runners, soccer players and weightlifters have been shown to possess higher BMD than non-active controls as well as suffer osteoporotic hip fractures at a significantly older age (Kettunen et al. 2010). This emphasises the potential of BMD to be maintained and the importance of weight-bearing exercise in contributing to skeletal integrity in later life.

Studying athletes who experience extreme amounts of loading can somewhat compensate for the aforementioned limitations associated with quantifying physical activity. Elite athletes in weight-bearing sports are a unique population who generally experience extreme amounts of mechanical loading, which, although not a perfect solution, presents an attractive model for future research studies hoping to investigate the impact of exercise on BMD. Additionally, by selecting homogeneous athlete groups, who compete in the same event to a similar standard, it would be reasonable to assume these individuals undertake similar training regimes/volumes. For instance, Billat et al. (2001) reported high-level male marathon runners with a personal best of < 2 h 16 min ran an average weekly distance of 168 km (± 20 km) and females with a personal best of < 2 h 36 min completed 150 km (± 17 km) on average.

### BMD, elite athletes and injury risk

Despite the benefits of weight-bearing activity for BMD, at the elite sporting level, too much activity to the point of overtraining can result in negative outcomes (Kuipers and Keizer 1988). A stress fracture would be one such outcome and is defined as a partial or complete fracture of bone from repeated application of force lower than that required to fracture a bone in a single loading (Iwamoto and Takeda 2003). Stress fracture injury occurs due to the repetitive mechanical loading that stimulates an incomplete remodelling response (Jones et al. 2002) and several factors are known to influence an individual’s susceptibility to experience a stress fracture (Bennell et al. 1999). Such factors include biomechanical gait (Milner et al. 2006), bone size and mechanical properties (Tommasini et al. 2005), nutritional factors (Nieves et al. 2010), training volume and rapid increments in volume (Snyder et al. 2006), small musculature and low BMD (Beck et al. 2000).

Unsurprisingly, higher incidence of lower limb stress fractures is observed in endurance runners in comparison with non-athletic controls. Significant amounts of site-specific loading combined with other factors typical of this group, such as low energy availability, can result in lower BMD and a higher risk of fracture occurrence (Loucks 2007). Stress fractures reportedly account for 50% of all injuries sustained by runners and military recruits, with higher incidence observed in females (Milner et al. 2006). However, there is a lack of research on stress fractures in running populations (Wright et al. 2015). Although lower BMD has been observed at the foot in female athletes with a history of stress fracture, compared to those without, this was accompanied by lower lean mass, leg-length discrepancy and fewer menstrual cycles per year, which may be influential (Bennell et al. 1996). Furthermore, determining accurate prevalence is also difficult due to the problematic nature of defining stress fractures. Significant misdiagnosis will occur unless limited to radiography, although this can still lack sensitivity and specificity (Wright et al. 2016).

Investigating BMD, with a particular emphasis on injury, is undoubtedly important because stress fractures have substantial implications for athletes. For instance, Marathon world record holder, Paula Radcliffe, reportedly suffered a stress fracture 3 months before the Beijing 2008 Olympics, limiting her preparation for and performance at that competition. Furthermore, Ranson et al. (2010) reported 43% of the elite fast bowlers they investigated developed symptomatic acute lumbar stress fractures in a 2-year follow-up period and subsequently missed 169 days of cricket, per episode, on average.

If athletes are unable to complete their desired or required training volume due to injury, this could have substantial negative effects on their performance and success. Additionally, if an athlete knows they may be susceptible to injury, this could be accounted for in their training programmes, by placing a greater emphasis on appropriate strengthening exercises and/or allowing longer rest periods between sessions. This valuable information for tailored training could then ultimately influence progression of athletes from amateur to elite or have implications for selection into high-level teams or sporting competitions. It is apparent that a substantial proportion of research in this area has been completed in military recruits (Wright et al. 2015). This is probably due to the ease of accessing large samples who undertake a quantifiable training load, as well as a desire to minimise waste of human and financial resources caused by injuries. However, it is difficult to directly extrapolate the findings of these military studies to elite runners due to differences in the level of physical fitness, footwear and the loads carried whilst running between these groups. Despite possible stress fractures, the positive benefits of physical activity/exercise on BMD in a broad population are evident. As discussed, there are a number of determinants influencing BMD but relatively little is known about the genetic influence on this phenotype and stress fractures, which could be pivotal for future understanding in both the sporting and public health domains.

## Genetic association with BMD

Although BMD is a multi-factorial phenotype, heritability of BMD is suggested to be 50–85% depending upon anatomical location (Ralston and Uitterlinden 2010). However, it must be emphasised that this proposed large genetic component is in a free-living population where most people will not complete extreme volumes of physical activity or be severely malnourished and thus, the influence of these other environmental factors on BMD will be reduced. Therefore, even a very substantial genetic contribution to BMD does not mean physical activity or other factors cannot notably affect an individual’s BMD (as shown in “BMD and physical activity”).

Due to this substantial genetic component, knowing the associated variants could be extremely beneficial for both functional research focus as well as application. For example, accuracy of fracture risk classification was improved by 7–10% at various sites in osteopenic patients by adding a genetic risk score from proposed common or rare variants associated with BMD and/or osteoporosis (Lee et al. 2014). In the future, this application might be utilised in athletic populations for risk stratification and injury prevention. However, utilising a genetic risk score with elite athletes is currently difficult due to a lack of known candidate genes associated with BMD in athletic populations, which emphasises the need for replication of potential candidate genes and specific studies on particular populations, who may possess high or low BMD, or demonstrate specific lifestyle choices/habits that influence BMD.

**Fig. 2:**
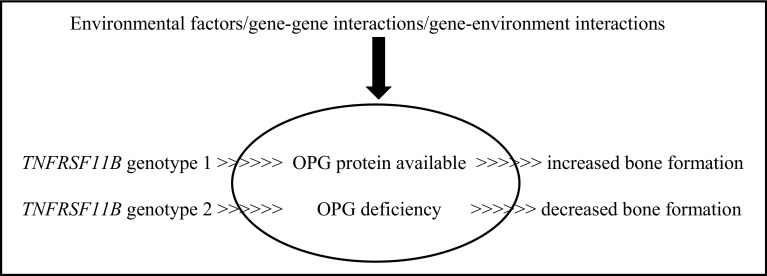
*TNFRSF11B* genotype influence on OPG availability and subsequent bone formation with the potential of environmental and interaction effects

Beginning in clinical populations, studies that selected candidate genes for association with BMD due to known biological function, such as *VDR*, insulin-like growth factor 1 (*IGF1*) and oestrogen receptor 1 (*ESR1*) (Gong and Haynatzki 2003), produced inconclusive findings. Candidate gene selection can be based on the premise that the protein plays a role in regulating bone cell function or calcium metabolism (Ralston and de Crombrugghe 2006), and the differing variants may affect bone mediators and consequently influence BMD. For example (as highlighted in Fig. 2 below), the human TNF receptor superfamily member 11b (*TNFRSF11B*) gene encodes the protein osteoprotegerin (OPG), which regulates bone resorption by inhibiting differentiation and activation of osteoclasts. OPG-deficient mice have been found to develop early-onset osteoporosis, and increased tissue mRNA expression has been observed in participants who possess specific haplotypes accompanied with reduced BMD, which may be due to increased expression resulting in stimulated osteoclast activity (Takács et al. 2010). This simplistic model forms the basis of genetic regulation on BMD but, in reality, the process is much more complex due to environmental factors and various kinds of interactions, which could have a substantial effect on gene expression and phenotype outcome. This potential impact of mechanical loading on gene expression can be understood by the substantial upregulation and downregulation of numerous genes following mechanical loading in rats (Mantila Roosa et al. 2011). Genes including FOS-like 1, AP-1 transcription factor subunit (*FOSL1*) and JunB proto-oncogene, AP-1 transcription factor subunit (*JUNB*) were both upregulated within 4 h after loading, whilst expression of Wnt/β-catenin signaling genes *SOST* and secreted frizzled-related protein 4 (*SFRP4*) was also altered in the synthetic phase of bone formation (Mantila Roosa et al. 2011). In the case of OPG, in vitro evidence demonstrated that compressive forces increase IL-6 and PGE_2_ production through the activation of intracellular calcium/extracellular signal-regulated kinase 1/2 and nuclear factor-κB translocation (Ca++/ERK1/2/NF-κB) signalling pathways, which results in decreased osteoblast OPG expression (and a decreased OPG/RANKL ratio) and enhanced matrix metallopeptidase (MMP) production, consequently increasing bone resorption (Sanchez et al. 2009).

Recent technological advances and large collaborations have seen a number of genome-wide association studies (GWAS) with BMD completed, which identified many more potential candidate genes and SNPs (Richards et al. 2012; Clark and Duncan 2015). However, the most prominent study to date, a meta-analysis conducted by Estrada et al. (2012), identified 56 loci associated with BMD, osteoporosis and/or fracture that accounted for ~ 6% of the variation in BMD. Overall, more than 66 genetic loci have been associated with (DXA derived) BMD via GWAS method, as well as many others through candidate gene association studies, and this number continues to increase, emphasising the extremely polygenic nature of BMD (Golchin et al. 2016). A further 153 loci have been associated with BMD estimated by quantitative ultrasound of the heel (Kemp et al. 2017). A specific recent addition, for instance, is a locus harbouring the Patched 1 (*PTCH1*) gene in an Icelandic population (Styrkarsdottir et al. 2016). This rapid discovery rate of new candidate genes and the fact many previously discovered candidate genes have had little or no replication through further study means only a very small number can be confidently suggested to have an association with BMD. Furthermore, the biological function or involvement with bone metabolism of 30 of these has yet to be elucidated and only 7 of the 66 have been associated in candidate gene studies previously or positively replicated afterwards (Hsu and Kiel 2012) although some have received no further study as of yet. To have only seven candidate genes positively associated through both methods so far is surprising, considering almost 100 different loci have been associated with BMD via a candidate gene approach (Hsu and Kiel 2012). Hsu and Kiel (2012) suggested a number of reasons why this may have occurred; first, false-negative findings due to the stringent level of statistical significance typically applied to GWAS data, or inadequate statistical power in some studies that were unable to replicate associations with modest effect sizes. On the other hand, false-positive findings of candidate gene association studies may have prevailed due to small sample sizes or publication bias (Munafo et al. 2004).

Additionally, strong gene–gene or gene–environment interactions could alter the number and identity of loci associated with BMD. This could apply to specific populations, such as athletes, due to the substantial influence of physical activity on BMD. Ultimately, this has resulted in few candidate genes emerging from GWAS and/or association studies that also have a known biological function relevant to bone. Therefore, further research using well-defined independent cohorts is needed to provide further evidence (Agueda et al. 2010). Clark and Duncan (2015) suggest greater use of “extreme cohorts” who might possess variants that have stronger associations with relevant phenotypes, which could include elite athletes at one end of a continuum (as mentioned in “BMD in athletic populations”) and osteoporotic individuals at the other. This approach has been applied to BMD successfully in a study of postmenopausal women with extremely high or low BMD, where GWAS revealed six novel genetic associations (Duncan et al. 2011).

Studies so far have only elucidated a small fraction of BMD variance and thus some of the unexplained heritability is likely due to a number of factors, including gene–environment interactions (Ackert-Bicknell and Karasik 2013). Despite the substantial effect of physical activity/exercise on BMD, there has been little research regarding gene–physical activity interactions and its effects on BMD in athletic populations. Therefore, this limited amount of research, as well as the variance in sample size and participant characteristics, means it is difficult to evaluate the extent of the gene–physical activity interaction with BMD or propose any definitive candidate genes that interact with environmental factors in determining BMD. However, looking at this relationship using specific cohorts or populations is gathering momentum—for example, investigations exploring interactions with others phenotypes, including obesity, are now being conducted (Marti et al. 2008). As mentioned previously (“BMD in athletic populations”), athletes would be an excellent sample group to explore this interaction as they present an extreme cohort regarding exercise undertaken and BMD.

### Genetic association with BMD and the relationship with physical activity

Mitchell et al. (2016) were the first to investigate the genetic influence on BMD and the relationship with physical activity using SNPs that had been associated with BMD using GWAS (Estrada et al. 2012). Analysis revealed physical activity interacted with ELKS/Rab6-interacting/CAST family member 1/Wnt family member 5B (*ERC1*/*WNT5B*) rs2887571 to influence bone mineral content in males and nominal interactions with physical activity were also observed with Wnt family member 16 (*WNT16*) rs3801387, axin 1 (*AXIN1*) rs9921222, *SOST* rs4792909 and stAR-related lipid transfer domain containing 3N-terminal-like (*STARD3NL*) rs6959212. Sclerostin has a negative effect on bone formation by inhibiting canonical Wnt signalling in osteoblasts and also stimulates osteoclastic bone resorption by increasing the RANKL/OPG ratio (via enhanced RANKL expression) (Appelman-Dijkstra and Papapoulos 2016). Despite this strong influence on bone metabolism, conflicting results regarding *SOST* variants and association with BMD have been reported in the literature (Sharma et al. 2015). Additionally, serum sclerostin concentration has been positively correlated with lumbar spine, femoral neck and total hip BMD but no variants were associated with BMD or sclerostin concentration (He et al. 2014). It is important to note that children/young adults (age 5–19 years) were the investigated cohort in the Mitchell et al. (2016) study. It is suggested that some BMD-associated loci may exert age-specific effects (Medina-Gomez et al. 2012), and thus the findings cannot be generalised to other populations.

Interesting findings have also been reported in candidate gene association studies. Kiel et al. (2007) discovered two SNPs in the LDL receptor-related protein 5 (*LRP5*) gene associated with differences in BMD, which were dependent upon volume of physical activity completed. The TT genotype of both the rs3736228 and rs2396862 SNPs was associated with lower BMD in more physically active men, but with higher BMD in less physically active men. Thus, the authors hypothesised that the substitution of a C with a T allele in the rs3736228 SNP could alter LRP5-mediated Wnt signalling in the case that the catabolic signals induced from the mechanical loading prevail over anabolic signalling. This was also the case when expressing alleles as a haplotype in vitro, where the T allele was associated with a decreased response to canonical Wnt3a signalling in comparison to the C allele. Activation of Wnt/β-catenin (canonical) signaling increases the sensitivity of osteoblasts to mechanical loading, which can occur via Wnt binding to low-density lipoprotein receptor-related proteins 5 and 6 co-receptors (Robinson et al. 2006; Krishnan et al. 2006). This mediation of Wnt signaling via different *LRP5* variants can both enhance and decrease BMD (Ferrari et al. 2005). Loss-of-function mutations in *LRP5* are also responsible for low bone mass disorders, such as osteoporosis pseudoglioma, whereas gain-of-function mutations have been suggested to cause high bone mass syndromes (Levasseur et al. 2005). Furthermore, *LRP5* variants, such as C135242T, have been associated with BMD variability in the general population (Koay et al. 2004) and ds2306862 in osteoporotic individuals (Mizuguchi et al. 2004), which highlights the strong influence *LRP5* may have on bone metabolism, particularly when considering a mechanical loading interaction.

Similar to some *LRP5* variants, the catechol-O-methyltransferase (*COMT*) val158met (rs4680) SNP has been reported to influence the association between physical activity and BMD suggesting that certain variants may be particularly important for BMD in individuals with low physical activity levels. Higher total BMD was observed in individuals completing greater levels of physical activity (> 4 h) compared to those undertaking lower activity (< 4 h) for GA and AA (lower enzyme activity) but not GG (higher enzyme activity) genotypes (Lorentzon et al. 2007). Although lower BMD was observed in the lower enzyme activity group, estradiol serum levels were not. COMT catalyses the methylation of catechol oestrogens to methoxy oestrogens (inactive metabolites) and thus lower COMT enzyme activity should result in less efficient inactivation of catechol oestrogens and higher BMD in these genotypes as has been shown in other studies (Eriksson et al. 2005). Therefore, a *COMT* genotype interaction may be present and the potential regulation of the BMD response to mechanical loading may be due to the involvement of oestrogen receptors as facilitators in a number of key pathways by which mechanical strain stimulates bone formation (Galea et al. 2013).

Interleukin 6 (*IL6*) is another potential candidate gene with a number of functional polymorphisms, suggested as candidates associated with BMD and/or osteoporosis. Meta-analysis revealed an association between the GG genotype in the *IL6* − 174G/C (rs1800795) polymorphism and low BMD, as well as increased risk of osteoporosis, in a Caucasian population (Ni et al. 2014). In the − 634C/G (rs1800796) polymorphism, the CC genotype was associated with greater BMD in Chinese pre-menarche girls who completed higher levels of physical activity (Li et al. 2008). Similarly, total body, lumbar spine and femoral neck BMD were lower in the GG genotype compared to the CC genotype by 0.03, 0.03 and 0.01 g/cm^2^, respectively, in an Asian population (*n* = 3068) following meta-analysis (Yan et al. 2015). IL-6 is primarily sourced in osteoblastic cells and increases interactions between osteoblasts and osteoclasts, thus stimulating bone resorption (Steeve et al. 2004). IL-6 is suggested to indirectly stimulate osteoclastogenesis by increasing RANKL gene expression in osteoblasts (Bakker and Jaspers 2015) and the G allele has been associated with elevated production and secretion of IL-6 in vitro (Kitamura et al. 2002). Therefore, the G allele and thus elevated IL-6 may be disadvantageous for bone density. Although there are limitations regarding control of other BMD-influencing variables and various cohorts used in these studies, *IL6* remains interesting, particularly when analysing a possible relationship with physical activity. In vitro studies have suggested IL-6 is produced by shear-loaded osteocytes and may influence bone mass by osteocytes reducing osteoblast activity via IL-6-mediated intercellular signalling (Bakker et al. 2014). Elevated IL-6 serum concentrations have also been observed in trained marathon runners immediately post-race, with a positive correlation between IL-6 concentration and running intensity (Ostrowski et al. 2000). In longitudinal studies, serum IL-6 concentration has been negatively associated with bone resorption and BMD in older adults although the literature is somewhat conflicting (Ding et al. 2008). *IL6* demonstrates the possibility of strong gene–environment interactions and studies that do not control for physical activity risk erroneous findings and/or results that are only applicable to limited portions of the population.

**Fig. 3:**
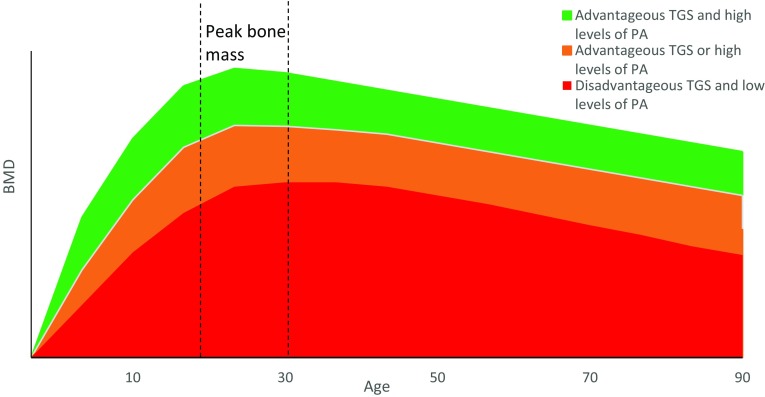
Schematic representation of typical age- and sex-related loss of BMD in men and the effect of physical activity and genetics

**Fig. 4:**
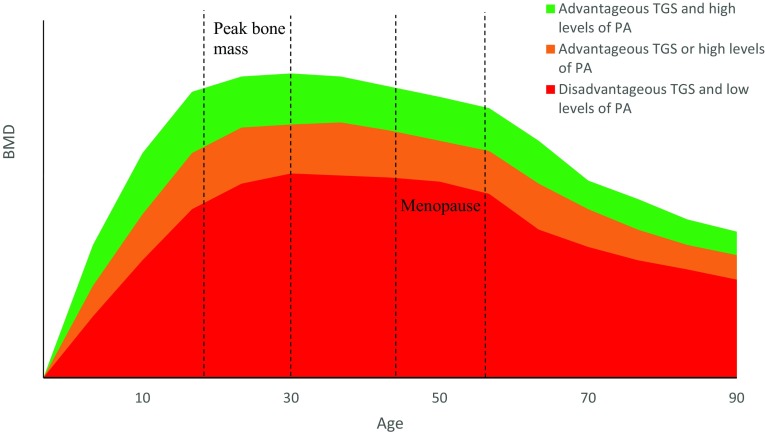
Schematic representation of typical age- and sex-related loss of BMD in women and the effect of physical activity and genetics

Overall, completing weight-bearing physical activity has been shown to increase BMD as discussed in “BMD and physical activity”. The effect of potential gene–physical activity interactions on BMD across the lifespan, however, has yet to be determined. It could be hypothesised that if an individual has a disadvantageous genetic profile and completes low levels of weight-bearing physical activity, they may be at risk for low BMD and potentially osteoporosis in later life (Disadvantageous TGS and low levels of PA) (Figs. 3 and 4). Those who may have a disadvantageous genetic predisposition, however, but complete sufficient weight-bearing activity to produce a substantial osteogenic response may be able to combat their negative genetic predisposition resulting in increased BMD, as evidenced in children (Mitchell et al. 2016) (Advantageous TGS or high levels of PA). Similarly, those who do not complete suitable levels of activity but possess an advantageous genetic profile may also present with moderate BMD (Advantageous TGS or high levels of PA). Those with an advantageous genetic profile who also complete large volumes of weight-bearing physical activity are likely to have the highest BMD (Advantageous TGS and high levels of PA), which could be induced from a gene–physical activity interaction.

**Fig. 5:**
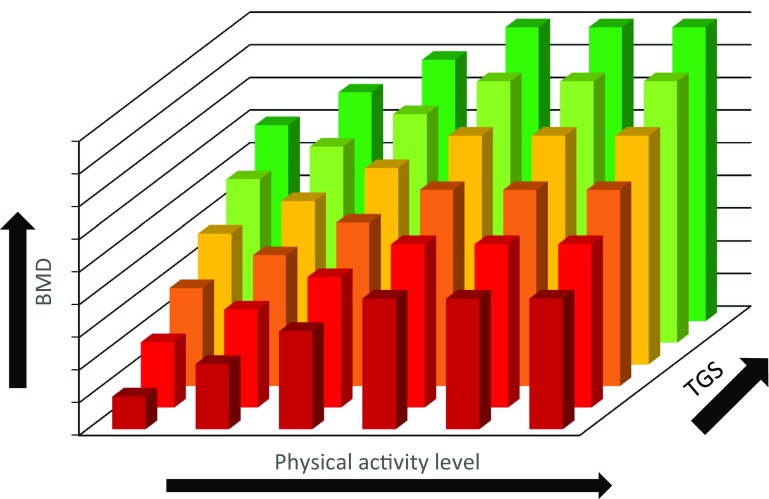
Schematic hypothetical representation of the BMD outcome for different individuals representing variable genetic profiles (TGS) and levels of physical activity

**Fig. 6:**
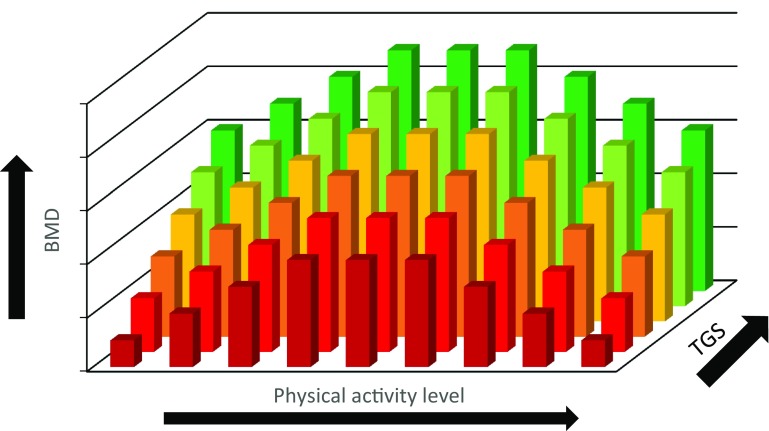
Schematic hypothetical representation of the BMD outcome for different individuals (e.g. endurance runners) representing variable genetic profiles (TGS) and levels of physical activity

In the case of a gene–physical activity interaction, a hypothetical relationship between genetics, physical activity and the resultant BMD is presented below (Fig. 5). Each bar represents a different individual and a hypothetical scenario for BMD ranging from a low BMD to a high BMD (the bar colour indicates BMD at any given level of physical activity in Figs. 5, 6). BMD is dependent on both genetics and physical activity level, so as physical activity level increases, BMD is enhanced for every individual regardless of their BMD before this increase in physical activity occurred. The magnitude of increase in BMD and maximum BMD level attained, however, are under the influence of genetics (Ralston and Uitterlinden 2010). Consequently, those with a more advantageous genetic predisposition, indicated by a higher total genotype score (TGS), combined with a higher volume of mechanical loading are more likely to reach a higher BMD than those with a disadvantageous genetic predisposition and/or a lower volume of mechanical loading, assuming all else is equal.

It is possible, however, that a linear relationship between physical activity dose and BMD response does not exist at the extremes of physical activity (PA). National Health and Nutrition Examination Survey (NHANES) data have previously demonstrated that BMD did not differ between males who reported completing four–six times more physical activity than the recommended guidelines (Whitfield et al. 2015). The physical activity and BMD relationship is still poorly understood and in the case of endurance runners, overtraining can negatively affect BMD (Fig. 6) due to the associated influence of energy availability. Other factors such as the type of activity and dietary intake, however, are also important in regard to the bone adaptation as discussed in “BMD in athletic populations” and would consequently affect this relationship.

### Genetic association with BMD in athletic populations

**Fig. 7:**
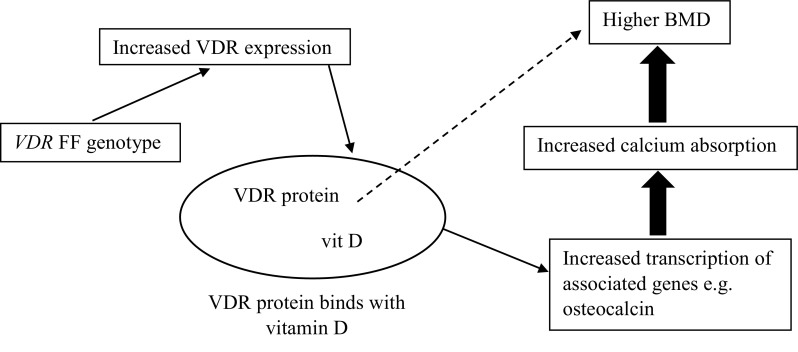
*VDR* rs2228570 FF genotype and the associated pathways leading to enhanced BMD

In 212 young males, significantly higher total body BMD in 84 weight-bearing athletes than 80 controls was observed in the FF (7.7%) and Ff (6.9%) but not ff (1.8%) genotypes of the *VDR* FokI rs2228570 polymorphism, whilst significantly lower total BMD was only observed in the FF (− 4.5%) genotype when comparing 48 swimmers with a control group (Nakamura et al. 2002b). This suggests that individuals with the FF genotype may be more responsive to mechanical loading, resulting in greater BMD when that environmental factor is prominent. This notion was further reinforced in 44 Japanese track and field athletes, where higher bone volume was expressed in those with the FF genotype, but not in those with the Ff genotype (Nakamura et al. 2002a). This particular polymorphism, Fokl (rs2228570), exhibits a C to T transition that creates an upstream initiation codon, leading to the production of VDR proteins that are three more amino acids in length. The F allele codes for the absence of the restriction, whilst the f allele codes for the presence of the initiation codon, which leads to the longer amino acid length (Gross et al. 1996; Ames et al. 1999). It is suggested that the F variant shows greater transactivation (protein expression) than the f variant and this increased biological activity (and associated increased intestinal absorption of calcium) could explain why higher BMD has been reported in those with the FF genotype (Arai et al. 1997; Colin et al. 2000; Uitterlinden et al. 2004; Ames et al. 1999) as detailed below (Fig. 7). *VDR* controls the transcription of other genes including bone gamma-carboxyglutamate protein/osteocalcin (*BGLAP*) that are instrumental for this calcium absorption and bone formation (Moran et al. 2014). A direct effect of osteoblastic/osteocytic VDR signalling on bone remodelling has also been proposed, although specific understanding of this notion is still lacking and largely depends on calcium balance (Lieben and Carmeliet 2013).

The potential association of *VDR* with BMD and/or fracture has also been supported across a number of different SNPs (rs1544410, rs7975232 and rs731236) in various cohorts, such as pre- and postmenopausal women (Riggs et al. 1995; Horst-Sikorska et al. 2007; Ji et al. 2010; Marozik et al. 2013). However, contradictory results have also been reported across these cohorts (Horst-Sikorska et al. 2013; Moran et al. 2015; Castelán-Martínez et al. 2015; Dabirnia et al. 2016). The highly conflicting nature of the findings may be due to not adjusting for covariates (e.g. BMI) as well as the different ethnic groups, sample sizes and study designs utilised (Xu et al. 2005).

A recent study of 99 elite academy footballers found a number of SNPs associated with bone phenotypes (trabecular density, cortical thickness and cross sectional area) using pQCT analysis. However, these associations were only observed before, but not after, a 12-week period of increased football training volume and thus no association between genotype and changes in bone parameters over time was observed. These variants included *SOST* rs1877632, *P2RX7* rs1718119, *P2RX7* rs3751143 as well as *TNFRSF11A* (RANK), *TNFSF11* (RANKL) and *TNFRSF11B* (OPG) SNPs rs9594738, rs1021188 and rs9594759 (Varley et al. 2018). Although no genotype–training interactions were observed for the SNPs analysed in this investigation, other candidate genes could be sensitive to physical loading (i.e. gene–environment interaction) and thus modulate athlete health (and, by extension, enhance endurance performance). Specifically, if an athlete has a genetic predisposition towards low BMD or elevated risk of stress fracture, exercise training and/or diet could be modified to accommodate.

### Genetic association with stress fracture injury

There is a lack of conclusive evidence regarding external determinants of stress fractures (Wright et al. 2015) as mentioned in “BMD, elite athletes and injury risk”. In more recent times, the idea of a proposed genetic influence has been investigated primarily in military recruits, due to the abrupt increase in training, large training volumes and high prevalence of stress fractures (Lappe et al. 2008). Examples have included the calcitonin receptor (*CTR*) rs1801197 and *LRP5* rs2277268 polymorphisms, which were associated with femoral neck stress fractures in 72 Finnish military recruits (Korvala et al. 2010). Participants who possessed the *CTR* C allele together with a *VDR* C-A haplotype were more protected from stress fractures, which may be due to the role of *CTR* in osteoclast-mediated bone resorption (Pondel 2000).

Furthermore, larger sized CAG androgen receptor (*AR*) gene repeats (> 16) were more common in Israeli military personnel who had suffered stress fractures (23%) than those who had not suffered this injury (13%) (Yanovich et al. 2011). A higher number of CAG repeats within the *AR* gene are inversely associated with the transcriptional response to testosterone (Zitzmann et al. 2001) and deficiency in such hormones could influence bone metabolism and potential bone loss (Mohamad et al. 2016; Khosla 2015).

Stress fracture susceptibility, in relation to genetics, has also been investigated in athletes for the first time recently, with findings suggesting that athletes with specific genetic variants may have an increased vulnerability to this injury (Varley et al. 2015, 2016, 2017). Interestingly, three of the same SNPs (*VDR* FokI rs2228570, *TNFSF11* rs1021188 and the loss of function *P2RX7* rs3751143) as mentioned above, alongside *TNFRSF11A* rs3018362, were associated with stress fracture incidence in the Stress Fracture in Elite Athlete (SFEA) cohort. However, a gain of function *P2RX7* SNP (rs1718119) was associated with multiple stress fracture occurrence. Functional expression of purinergic receptor P2X7 primarily regulates configuration of osteoclasts (Agrawal et al. 2010), as well as augmenting bone formation via a cell-autonomous role that leads to stimulation of mineralisation (Panupinthu et al. 2008), which may explain why some *P2RX7* polymorphisms have also been associated with low baseline and accelerated bone loss in post-menopausal women (Gartland et al. 2012). *P2RX7* is a particularly interesting candidate gene in regard to potential gene–physical activity interactions and outcomes for BMD. Mice with a null mutation of *P2RX7* have been reported to show > 73% reduced sensitivity to mechanical loading (Li et al. 2005). Fluid shear stress increased prostaglandin (PG)E_2_ release in wild-type osteoblast cells but had no effect on PGE_2_ release in knockout osteoblast cells. PGE_2_ administration activates cortical bone modelling resulting in increased bone mass (Jee et al. 1990) and Li et al. (2005) suggested that these findings indicate ATP signalling through *P2RX7* is important for mechanically induced release of prostaglandins by bone cells and subsequent bone formation. Consequently, variation in *P2RX7* SNPs such as rs3751143 could result in differing responses to mechanical loading and alterations to BMD, potentially influencing stress fracture susceptibility.

Although research investigating genetic influence on stress fracture has begun using the SFEA cohort, this was a loosely defined group, which comprised athletes of mixed abilities and from a range of sports. A more focussed approach, which removes the variability (i.e. loading/training patterns) introduced by incorporating athletes from different sports into one investigation, would be advantageous.

## Future directions and conclusions

There are numerous polymorphisms that need further exploration vis-à-vis BMD. In particular, gene–environment (i.e. gene–physical activity) interactions are likely to contribute substantially to inter-individual differences in BMD throughout the human lifespan. Exciting findings have been observed in regard to gene–physical activity interactions and genetic associations with stress fracture, particularly in variants of pathways involved in the adaptation of bone to mechanical loading, such as the RANK/RANKL/OPG system.

Therefore, the study of specific cohorts, who experience unusually high mechanical loads and who may display unusual bone phenotypes and/or possess genetic characteristics that differ from the norm, may provide novel insight into the area. Such individuals include elite athletes, who are at the extremes of human physiological capability, experience much greater environmental (mechanical) stress than most and might possess a genotype particularly suitable to tolerate those stresses.

GWAS or ideally whole genome sequencing (WGS) studies using athletic populations with their differentiating extreme phenotypes are, in principle, the next logical steps to identify key polymorphisms. Detailed study of gene function can follow. However, most GWAS designs cannot account for gene–gene/gene–environment interactions and only analyse SNPs with minor allele frequencies of more than 1%, not rare variants that may lie between 0.1–1% or even lower. Thus, GWAS is appropriate for the discovery of common variants that may confer low/moderate risk but are underpowered for the detection of rare variants, which may have a large influence on a complex phenotype according to the common disease/rare variant hypothesis (Li and Leal 2008). Conducting GWAS or WGS studies is also extremely challenging due to the associated costs and difficulty in recruiting sufficiently large numbers of such a specific population. Even a panel of SNPs for investigation that is far lower in number than used in contemporary GWAS, for example, 500 SNPs, would require a sample size of 1200 to detect an effect size of 0.02 in a continuous trait, assuming 80% statistical power, a minor allele frequency of 20% and an alpha level of 0.0001. Approximately the same size of sample would be needed for each group of a case–control study design, assuming the same parameters and an effect size (odds ratio) of up to 1.4 (Bouchard 2011).

While the large cohorts necessary for GWAS and eventually WGS studies of BMD in athletes are built, smaller samples (steps towards building the bigger sample) can be used to test hypotheses about genetic variants emerging from GWAS in relevant clinical populations. Assessing bone and injury phenotype data in those athletes will also enhance understanding of any observed genotype–phenotype relationship (Wang et al. 2013). A relatively homogenous group of athletes who experience high mechanical loads on some bone structures, such as endurance runners, would be suitable for this kind of investigation. Specifically, measuring areal BMD via DEXA scanning, with a particular emphasis on the primary loading sites in this population, would probably provide appropriate data to combat some of the challenges identified in this review. It would be fascinating to discover whether those athletes have a genotype that enhances BMD, protects against the effects of the large volume of training required and reduces risk of stress fracture. One preliminary report (using just 14 participants) even documents an attempt to reduce the risk of tendon, ligament and bone injuries by modifying athlete training programmes based upon genetic characteristics (Goodlin et al. 2015). This illustrates the kinds of future applications possible in this field *after* the more fundamental research has been conducted successfully.

## Abbreviations

AXIN1: Axin 1
BMD: Bone mineral density
BGLAP: Bone gamma-carboxyglutamate protein
CA++: Intracellular calcium
CTR: Calcitonin receptor
COMT: Catechol-*O*-methyltransferase
ERC1: ELKS/Rab6-interacting/CAST family member 1
ERK1/2: Extracellular signal-regulated kinase 1/2
ESR1: Oestrogen receptor 1
FOSL1: FOS-like 1, AP-1 transcription factor subunit
GWAS: Genome-wide association studies
IGF1: Insulin-like growth factor 1
IL6: Interleukin 6
JUNB: JunB proto-oncogene, AP-1 transcription factor subunit
LRP5: LDL receptor-related protein 5
MMPs: Matrix metallopeptidases
NHANES: National Health and Nutrition Examination Survey
NF-κB: Nuclear factor-κB translocation
OPG: Osteoprotegerin
PGE_2_: Prostaglandin E_2_
PTCH1: Patched 1
P2RX7: Purinergic receptor P2X7
RANK: Receptor activator of nuclear factor κB
RANKL: Receptor activator of nuclear factor κB ligand
SFRP4: Secreted frizzled-related protein 4
SNP: Single nucleotide polymorphism
SOST: Sclerostin
STARD3NL: StAR-related lipid transfer domain containing 3 N-terminal like
TNFSF11: TNF receptor superfamily member
TNFRSF11A: TNF receptor superfamily member 11a
TNFRSF11B: TNF receptor superfamily member 11b
VDR: Vitamin D (1,25-dihydroxyvitamin D_3_) receptor
WNT5B: Wnt family member 5B
WNT16: Wnt family member 16
